# Isolation, characterization, and comparative genomic analysis of a phage infecting high-level aminoglycoside-resistant (HLAR) *Enterococcus faecalis*

**DOI:** 10.7717/peerj.9171

**Published:** 2020-05-21

**Authors:** Danial Nasr Azadani, Daiyuan Zhang, J. Robert Hatherill, David Silva, Jeffrey W. Turner

**Affiliations:** 1 Life Sciences, Texas A&M University—Corpus Christi, Corpus Christi, TX, United States of America; 2 Natural Sciences, Del Mar College, Corpus Christi, TX, United States of America

**Keywords:** Phage, Enterococcus, *E. faecalis*, Antibiotic resistance, Diversity, Genomics, Pangenome

## Abstract

*Enterococcus* is a genus of Gram-positive bacteria that are commensal to the gastrointestinal tracts of humans but some species have been increasingly implicated as agents of nosocomial infections. The increase in infections and the spread of antibiotic-resistant strains have contributed to renewed interest in the discovery of *Enterococcus* phages. The aims of this study were (1) the isolation, characterization, and genome sequencing of a phage capable of infecting an antibiotic-resistant *E. faecalis* strain, and (2) the comparative genomic analysis of publicly-available *Enterococcus* phages. For this purpose, multiple phages were isolated from wastewater treatment plant (WWTP) influent using a high-level aminoglycoside-resistant (HLAR) *E. faecalis* strain as the host. One phage, phiNASRA1, demonstrated a high lytic efficiency (∼97.52%). Transmission electron microscopy (TEM) and whole-genome sequencing (WGS) showed that phiNASRA1 belongs to the Siphoviridae family of double-stranded DNA viruses. The phage was approximately 250 nm in length and its complete genome (40,139 bp, 34.7% GC) contained 62 open reading frames (ORFs). Phylogenetic comparisons of phiNASRA1 and 31 publicly-available *Enterococcus* phages, based on the large subunit terminase and portal proteins, grouped phage by provenance, size, and GC content. In particular, both phylogenies grouped phages larger than 100 kbp into distinct clades. A phylogeny based on a pangenome analysis of the same 32 phages also grouped phages by provenance, size, and GC content although agreement between the two single-locus phylogenies was higher. Per the pangenome phylogeny, phiNASRA1 was most closely related to phage LY0322 that was similar in size, GC content, and number of ORFs (40,139 and 40,934 bp, 34.77 and 34.80%, and 60 and 64 ORFs, respectively). The pangenome analysis did illustrate the high degree of sequence diversity and genome plasticity as no coding sequence was homologous across all 32 phages, and even ‘conserved’ structural proteins (e.g., the large subunit terminase and portal proteins) were homologous in no more than half of the 32 phage genomes. These findings contribute to a growing body of literature devoted to understanding phage biology and diversity. We propose that this high degree of diversity limited the value of the single-locus and pangenome phylogenies. By contrast, the high degree of homology between phages larger than 100 kbp suggests that pangenome analyses of more similar phages is a viable method for assessing subclade diversity. Future work is focused on validating phiNASRA1 as a potential therapeutic agent to eradicate antibiotic-resistant *E. faecalis* infections in an animal model.

## Introduction

*Enterococcus* is a diverse genus of Gram-positive bacteria and a component of the human gastrointestinal microflora (Murray, 1990). Most species and strains are commensal but a minority are human pathogens (Fisher & Phillips, 2009). Namely, in the past 30 years, a growing number of nosocomial infections have been attributed to *E. faecalis* and *E. faecium* (Moellering, 1992; Guzman Prieto et al., 2016). Moreover, antibiotic-resistant strains have been increasingly associated with nosocomial bacteremia, surgical wound infections, and urinary tract infections (Gilmore, Lebreton & Van Schaik, 2013; Lebreton et al., 2013).

*Enterococcus* infections are routinely treated with aminoglycosides (e.g., gentamicin and streptomycin) in combination with a cell wall inhibitor like ampicillin (Moellering, 1971). However, clinical *E. faecalis* and *E. faecium* isolates are frequently resistant to both aminoglycosides and cell wall inhibitors (Lebreton et al., 2013; Guzman Prieto et al., 2016). Mechanisms of resistance to aminoglycosides include mutation of the 30S ribosomal subunit and the acquisition of aminoglycoside modifying enzymes (AMEs) via horizontal gene transfer (HGT), while mechanisms of resistance to cell wall inhibitors include the mutation of penicillin-binding proteins and the HGT-mediated acquisition of B-lactamases (Hollenbeck & Rice, 2012).

A common theme in the evolution of antibiotic resistance is that long-term exposure to low levels of antibiotics (i.e., below the minimum inhibitory concentration, MIC) can lead to high-level resistance (Wistrand-Yuen et al., 2018). The excretion of antibiotics by patients makes wastewater a source of low-level antibiotic exposure (Khan, Söderquist & Jass, 2019), and urban wastewater treatment plants have been identified as hotspots for antibiotic-resistant bacteria (Rizzo et al., 2013). Previous studies have shown that a wide range of pharmaceuticals, including antibiotics, can be detected in wastewater (Kostich, Batt & Lazorchak, 2014), and *Enterococcus* exhibiting high-level resistance to aminoglycosides and cell wall inhibitors have been isolated from wastewater (Luczkiewicz et al., 2010).

In response to the emergence and spread of antibiotic-resistant bacteria, the World Health Organization (WHO), citing data from 129 member states, concluded that antibiotic resistance is an alarming threat to public health in every region of the world (WHO, 2014). The United States Centers for Disease Control and Prevention (CDC) released a similar report that detailed the incidence, death, and costs of antibiotic-resistant infections per year in the United States: 2,049,442 illnesses, 23,000 deaths, and $20 billion (US) in healthcare spending (CDC, 2013). Together, these two reports highlight the growing challenge of combating resistance and both encourage the development of new antimicrobial treatments.

The global decline in the effectiveness of antibiotics has renewed interest in using phage to treat antibiotic-resistant infections (Lin, Koskella & Lin, 2017). The potential for phage-based treatment was recognized by Felix d’Herelle and Giorgi Eliava in 1923 but the subsequent discoveries of penicillin by Alexander Fleming in 1928 and sulfonamide by Gerhard Domagk in 1932 shifted focus toward the development and production of antibiotic therapies (Zaffiri et al., 2012). Regardless, the practice of phage therapy continued (Thiel, 2004; Lin, Koskella & Lin, 2017) and recent studies have confirmed the utility of phage in the treatment of antibiotic-resistant infections including *Enterococcus* infections (Biswas et al., 2002; Cheng et al., 2017).

Knowledge of phage biology and diversity remains limited. For example, although the first phage genome phiX174 (Sanger et al., 1977) was sequenced 20 years prior to the first bacterial genome *Haemophilus influenzae* (Fleischmann et al., 1995), public databases contain relatively few phage genomes (e.g., the National Center for Biotechnology Information (NCBI) databases contained 9,220 phage genomes versus 202,218 bacterial genomes at the time of this submission). In the example of *Enterococcus*, NCBI databases contained only 38 *Enterococcus* phage genomes versus 3,564 *Enterococcus* genomes. Expanding knowledge of phage biology and diversity is, therefore, a critical need. Here, we report the isolation of an *Enterococcus* phage, sourced from wastewater, capable of infecting a high-level aminoglycoside-resistant (HLAR) *E. faecalis* strain. The characterization of this phage included culture-based and comparative genomic analyses aimed at expanding knowledge of *Enterococcus* phage biology and diversity.

## Material and Methods

### Bacterial strains and growth conditions

*E. faecalis* ATCC 49533 (American Type Culture Collection, Manassas, VA, USA) was used as the phage host. The strain’s high-level resistance to streptomycin was described previously (Spiegel, 1988). A lyophilized stock was revived by growing overnight (18 h) in brain heart infusion (BHI) broth (Becton Dickinson, Franklin Lakes, NJ, USA) at 37 °C with shaking (120 rpm). Cells were cryopreserved by mixing overnight culture and 50% glycerol at a 1:1 ratio (25% final concentration) and immediately freezing at −80 °C. When required, the frozen cell stock was streaked on BHI agar plates (BHI broth supplemented with 1% agar) and a single isolated colony was inoculated and cultured in BHI broth overnight (18 h) at 37 °C with shaking (120 rpm).

### Sample collection and phage isolation

The following method was adapted from a broadly-implemented, course-based undergraduate research program (CURE): Science Education Alliance-Phage Hunters Advancing Genomics and Evolutionary Science (SEA-PHAGES) (Jordan et al., 2013). A 1 L sample of influent was collected at the Greenwood wastewater treatment plant (WWTP) in Corpus Christi, Texas, USA. The sample was placed on ice (4 °C), transported to the laboratory, and processed within one hour. A 20 mL subsample was enriched in 20 mL of 2X BHI broth supplemented with five mL of AD supplement (a 0.22 µm filter-sterilized solution of 145 mM NaCl with 5.0% albumin and 2.0% glucose), 200 µL of 100 mM CaCl_2_ (1 mM final concentration), and five mL of mid-exponential phase *E. faecalis* ATCC 49533 in a 250 mL baffled flask for 18 h (37 °C, 120 rpm). A 25 mL aliquot of the enriched sample was centrifuged at 2,000×g for 10 min to pellet bacteria and particulate organic matter. The phage lysate was obtained by filtering five mL of the supernatant through a 0.22 µm low protein binding polyethersulfone syringe filter (Fisher Scientific, Waltham, MA, USA). The phage lysate was then serially diluted using SM buffer (Teknova, Hollister, CA, USA). The host was infected with the phage lysate by adding 50 µL of each dilution to 500 µL of mid-exponential phase *E. faecalis* ATCC 49533. The resulting host-phage mixture was incubated at room temperature (25 °C) for 15 min. A plaque assay was carried out by adding 4.5 mL of molten (55 °C) BHI top agar (0.5% agar) to each phage-host mixture and then applied onto warm (37 °C) BHI base agar plates, which were incubated overnight (24 h) at 37 °C. The following day, a single plaque from the 10^−3^ dilution plate was streaked onto a BHI agar plate, and a mixture of molten (55 °C) BHI top agar (4.5 mL) and mid-exponential phase host (0.5 mL) was poured on the streaked plate, followed by incubation overnight (24 h) at 37 °C. This streak test was repeated three times to purify the single phage. All BHI agar plates used in the plaque assay were supplemented with 1 mM CaCl_2_ and 1 mM streptomycin (final concentration).

### Phage morphology

Transmission electron microscopy (TEM) imaging of the phage was completed at the University of North Texas Center for Advanced Research and Technology, Denton, Texas, USA. Briefly, one mL of HTL (High titer lysate 1×10^8^ PFU mL^−1^) was centrifuged at 10,000×g, 4 °C for one hour to pellet the phage. The pellet was re-suspended in 100 µL of fresh phage buffer and incubated on ice for another hour. A 10 µL aliquot of the phage suspension was applied on a copper grid for five minutes and any excess liquid was wicked away with a filter paper. The grid was then washed twice by adding 10 µL of ultrapure water, which was allowed to sit for two minutes before drying with a filter paper. Lastly, 10 µL of 1.0% uranyl acetate was added to negatively stain the grid for two minutes and the excess stain was wicked away with a filter paper before air-drying for five minutes. Electron micrographs were taken with a Tecnai G2 F20 S-Twin 200 keV field emission scanning transmission electron microscope (S/TEM) (FEI Company, Hillsboro, OR, USA) with precision electron diffraction (PED).

### Phage infectivity

The burst size was determined as described previously (Ellis & Delbrück, 1939) with modification. Briefly, the phage titer was measured at 1.34 × 10^9^ plaque-forming units (PFU) mL^−1^ by adding serial dilutions of phage lysate to lawns of the *E. faecalis* ATCC 49533 host. Next, 100 µL of the phage lysate was added to one mL of mid-exponential phase host (OD_600_ = 0.8; multiplicity of infection [MOI] = 0.01). The host-phage mixture was incubated in a 37 °C water bath for five minutes to allow phage attachment. After incubation, the mixture was centrifuged at 4,800× g for 5 min at 4 °C. The supernatant was removed and the pellet was suspended 10 mL of BHI broth. Subsamples (100 µL) subsamples were taken at 5-minute intervals for 85 min and used for titer measurement by double-layer agar plating. The phage titer was plotted against time to measure the burst size.

### Efficiency of lysogeny

To estimate the lysogenic efficiency, 100 µL of phage lysate (1.34 × 10^9^ PFU mL^−1^) was plated on five BHI agar plates. Concurrently, a mid-exponential phase (OD_600_ = 0.8) culture of *E. faecalis* ATCC 49533 was serial diluted and 100 µL of the 10^−5^ to 10^−9^ dilutions were plated on the phage seeded plates. The negative control was created by plating the same host dilutions on five unseeded plates. Plates were incubated at 37 °C for 96 h. The lysogenic efficiency was calculated by dividing the CFU (colony forming unit) *E. faecalis* on seeded plates by the CFU *E. faecalis* on unseeded plates and multiplying the quotient by 100 (Equation A). Conversely, the lytic efficiency was equal to 100% minus the percentage of lysogeny (Equation B).

\begin{eqnarray*}\text{Equation}~\mathrm{A}: \frac{\text{CFU seeded plates}}{\text{CFU unseeded plates}} \mathrm{X}~100& =\text{Lysogenic efficiency} (\text{%}) \end{eqnarray*}

\begin{eqnarray*}\text{Equation}~\mathrm{B}:100\text{%} - \text{Lysogenic efficiency} (\text{%})& =\text{Lytic efficiency} (\text{%}) \end{eqnarray*}

### Host specificity

To evaluate the interspecies host range, the phage was tested against other Gram-positive cocci hosts: *E. faecium* ATCC 19434*, Staphylococcus* aureus (Cat. #155554A, Carolina Biological Supply Company, Burlington, NC, USA) and *Staphylococcus saprophyticus* (Cat. #851031, Ward’s Science, Rochester, NY, USA). Intraspecies host range was tested against several *E. faecalis* hosts: five clinical strains obtained from ATCC and 12 environmental strains obtained from a concurrent bacterial source-tracking study in Corpus Christi Bay (Turner et al., 2018a) (Table 1). Briefly, 500 µL of mid-exponential phase host was added to 4.5 mL of molten (55 °C) BHI top agar, which was then poured on top of BHI agar plates. The plates were allowed to solidify at room temperature (23 °C) before spot testing with 10 µL of a new phage lysate (1.2 ×10^7^ PFU mL^−1^ MOI 0.1). Plates were then incubated for 24, 48, 72, and 96 h at 37 °C and inspected for zones of lysis.

**Table 1:** Infectivity of phiNASRA1 against various host strains.

Host strain	Infectivity^a^
*E. faecalis* 2.1	–
*E. faecalis* 2.19	–
*E. faecalis* 11.1	–
*E. faecalis* 11.19	–
*E. faecalis* 11.24	–
*E. faecalis* 13.1	–
*E. faecalis* 13.16	+
*E. faecalis* 13.25	–
*E. faecalis* 13.28	–
*E. faecalis* 16.4	–
*E. faecalis* 16.26	–
*E. faecalis* 16.33	–
*E. faecalis* ATCC 29212	–
*E. faecalis* ATCC 33186	+
*E. faecalis* ATCC 49533	+
*E. faecalis* ATCC 51299	–
*E. faecalis* ATCC 51575	–
*E. faecium* ATCC 19434	–
*S. aureus* 155554A	–
*S. saprophyticus* 851031	–

**Notes.**

a Strains 33186 and 49533 showed infectivity within 24 h while 13.16 showed infectivity within 48 h.

### Antibiotic susceptibility

The susceptibility of all strains (*N* = 20) to streptomycin was determined in triplicate as described previously (Turner et al., 2018b). Briefly, strains were grown overnight (18 h at 37 °C) on Mueller-Hinton (MH) agar plates (Becton, Dickinson and Company, Franklin Lakes, NJ, USA). The overnight culture was diluted in sterile 0.45% NaCl until the turbidity approximated a 0.5 McFarland standard. The diluted culture was used to seed a bacterial lawn on freshly prepared MH plates using a sterile cotton swab. Plates were divided into quadrants and three streptomycin discs (10 µg) and one negative control (the NaCl solution) were placed in the center of the quadrants. Plates were then incubated overnight (18 h at 37 °C) and zones of inhibition were measured and interpreted according to the Clinical and Laboratory Standards Institute (CSLI) guidelines. *E. faecalis* ATCC 49533 (Str^R^) was used as the positive control.

### Genome sequence

Genomic DNA was isolated from the phage lysate (1.34 ×10^9^ PFU mL^−1^) using a Wizard DNA Purification Kit (Promega, Madison, WI, USA) according to the manufacturer’s protocol. The DNA was sequenced at the North Carolina State Genomic Sciences Laboratory (Raleigh, NC, USA) on an Illumina MiSeq instrument using 150 bp single-read chemistry. The sequencing facility processed the raw reads and assembled the genome using the CLC Genomics Workbench version 6.5.1 (Qiagen, Valencia, CA, USA). Briefly, raw reads were processed for quality (limit = 0.05), ambiguous bases (maximum 2 ambiguous bases allowed per read), Illumina adapters, and length (150 bp cutoff). The complete genome was then assembled de novo using a length fraction of 0.7 and a similarity fraction of 0.9 with default word and bubble sizes. The phage termini and packaging mechanism was determined using PhageTerm (Garneau et al., 2017) in the Texas A&M University Center for Phage Technology (CPT) Galaxy server. The phage genome sequence that was reorganized base on termini positions was used for annotation in RAST using settings optimized for bacteriophage (McNair et al., 2018).

### Phylogenetics

The relatedness of 32 publicly-available *Enterococcus* phage with complete genome sequences, including phiNASRA1 (isolated in this study), was assessed by constructing maximum-likelihood (ML) trees based on the large subunit terminase and portal proteins. A detailed description of those 32 phages, including provenance, was provided in Table S1. Protein sequences were recovered from Genbank files that were downloaded from NCBI (see Table S1 for accession numbers). The sequences were aligned individually with MUSCLE version 3.6 (options -stable -maxiters 1 -diags) (Edgar, 2004), trimmed with trimAL version 1.2 (options -noallgaps) (Capella-Gutiérrez, Silla-Martínez & Gabaldón, 2009), and ML trees were constructed with IQ-TREE version 1.5.5 (options -st AA -nt AUTO -v -m TEST -bb 1000) (Nguyen et al., 2015) with 1,000 ultrafast bootstraps (Minh, Nguyen & Haeseler von, 2013) using the best fit model as determined by ModelFinder (Kalyaanamoorthy et al., 2017). The trees were illustrated with FigTree version 1.4.3 (http://tree.bio.ed.ac.uk/software/figtree/).

### Pangenome analysis

The relatedness of the above 32 publicly-available *Enterococcus* phages, including phiNASRA1, was also assessed by conducting a pangenome analysis. This analysis was completed with GET_HOMOLOGUES version 3.2.1 (options -M -t 0 -e 1 -r phiNASRA1.gbk) (Contreras-Moreira & Venuesa, 2013) and the compare_clusters.pl script was used to compute a binary pangenome matrix. The resulting pangenome binary matrixes were passed to IQ-TREE version 1.5.5 (options -st BIN -nt AUTO -v -m TEST -bb 1000) to construct an ML tree with 1,000 ultrafast bootstraps as described above. A more targeted pangenome analysis of phage larger than 100 kbp (ECP3, EFLK1, EFDG1, and phiEF24C) was completed with GET_HOMOLOGUES version 3.2.1 (options -M -t 0 -e 1 -r EFDG1.gbk).

## Results

### Phage isolation and morphology

**Figure 1:**
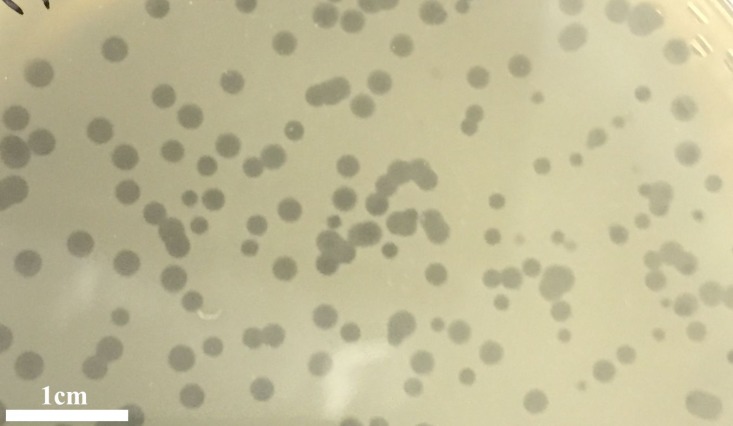
Plagues. Plaques of the phage phiNASRA1 on BHI agar. The mean diameter of the plaques was approximately three mm.

**Figure 2:**
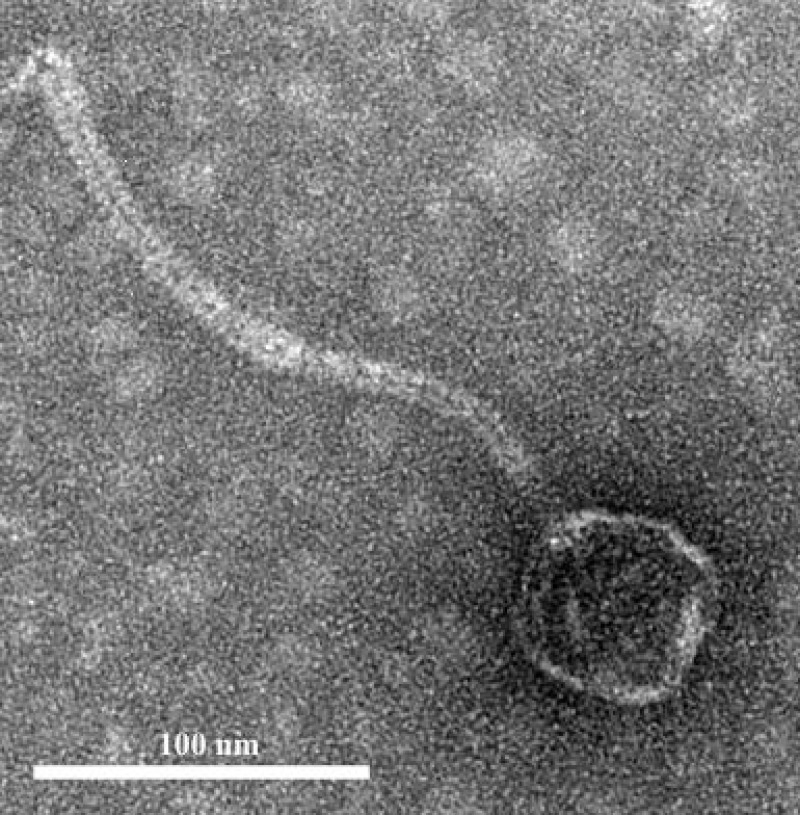
Phage morphology. Transmission electron microscope** (**TEM) image of phage phiNASRA1 showing the T7 symmetry with non-enveloped icosahedron head (∼60 nm in diameter) and a non-contractile tail (240 nm in length).

The infection of the *E. faecalis* ATCC 49533 host with WWTP phage lysate resulted in the formation of clear plaques, approximately three mm in diameter, as shown in Fig. 1. Within this population of phages, a small number of opaque plagues demonstrated morphological variability. A single phage was purified by multiple streak tests and named phiNASRA1. TEM imaging of the phage revealed a non-enveloped icosahedron head (∼60 nm in diameter) and a non-contractile tail (∼240 nm in length) (Fig. 2). Following the guidelines established by the International Committee of Taxonomy of Viruses (ICTV), phiNASRA1 was classified as a double-stranded DNA virus of the Siphoviridae family in the order Caudovirales.

### Phage infectivity

**Figure 3:**
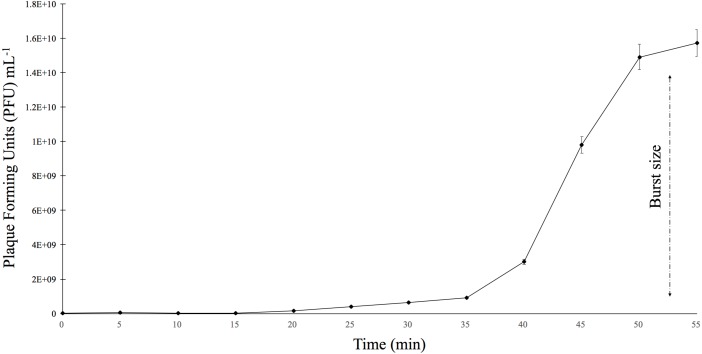
One-step growth curve. The one-step growth curve of phage phiNASRA1 showing the growth of plaque-forming units (PFU) over time. The standard error bars represent the variance between the two replicates.

The burst size was determined using *E. faecalis* ATCC 49533 as the host and a multiplicity of infection (MOI) of 0.01 (Fig. 3). The growth curve showed that phiNASRA1 exhibited a burst size of approximately 1.43 ×10^10^ PFU mL^−1^ (Fig. 3). In a separate experiment, the lytic efficiency was calculated as 97.52%, meaning that all but 2.48% of host cells were lysed during the 96-hour incubation.

### Host specificity

When testing interspecies host range, phiNASRA1 did not infect *E. faecium* ATCC 19434*, S. aureus* 155554A, or *S. saprophyticus* 851031. When testing intraspecies host range, phiNASRA1 infected three *E. faecalis* strains (13.16, ATCC 33186, and the original ATCC 49533 host) (Table 1).

### Antibiotic susceptibility

All strains exhibited resistance to streptomycin with one exception: *E. faecalis* 11.1 exhibited an intermediate level of susceptibility.

### Genome sequence

The genome sequencing of phiNASRA1 generated 636,206 raw reads. The complete genome was comprised of a single contig 40,139 bp in length (average coverage of 475.5X) and 34.7% GC content, and the RAST-based annotation detected 62 open reading frames (ORFs) (Table 2). Thirty-three of the ORFs encoded hypothetical proteins while the remaining 29 encoded proteins with known functions including capsid scaffolding, capsid morphogenesis, transcriptional regulation, and DNA replication, but no tRNAs or drug resistance genes were detected. The PhageTerm analysis revealed that phiNASRA1 has 3′ cohesive ends with a HK97-like packaging mechanism. The phiNASRA1 whole-genome shotgun project has been deposited at DDBJ/ENA/GenBank under the accession number MG264739.

**Table 2:** Detailed summary of the open reading frames (ORFs) detecting in phiNASRA1.

**ORF**	**Start**	**End**	**Strand**	**Nucleotide**	**Size(aa)**	**Start Codon**	**Predicted Function**
1	485	114	–	372	124	ATG	Phage-associated homing endonuclease
2	682	485	–	198	66	ATG	Phage protein
3	964	761	–	204	68	ATG	Hypothetical protein
4	1467	1910	+	444	148	ATG	Hypothetical protein
5	1923	2330	+	408	136	ATG	Phage protein
6	2330	2959	+	630	210	ATG	Phage tail tape measure protein
7	3005	3103	+	99	33	ATG	Hypothetical protein
8	3128	3273	+	156	52	ATG	Hypothetical protein
9	3270	3410	+	141	47	ATG	Hypothetical protein
10	3410	3634	+	225	75	ATG	Hypothetical protein
11	3634	3846	+	213	71	ATG	Hypothetical protein
12	3843	4061	+	219	73	ATG	Hypothetical protein
13	4058	4297	+	240	80	ATG	Hypothetical protein
14	4586	6166	+	1581	527	GTG	Hypothetical protein
15	6255	6698	+	444	148	ATG	Hypothetical protein
16	6746	7027	+	282	94	ATG	Hypothetical protein
17	7020	7190	+	171	57	ATG	Hypothetical protein
18	7192	7401	+	210	70	ATG	Hypothetical protein
19	7404	7640	+	237	79	ATG	Hypothetical protein
20	7624	7983	+	360	120	ATG	Hypothetical protein
21	7976	9271	+	1296	432	ATG	Hypothetical protein
22	9337	9534	+	198	66	ATG	Hypothetical protein
23	9547	10287	+	741	247	ATG	Hypothetical protein
24	10241	10750	+	510	170	ATG	NHN homing endonuclease
25	10761	10967	+	207	69	ATG	Hypothetical protein
26	11124	11570	+	447	149	ATG	Hypothetical protein
27	11572	11570	+	207	69	ATG	Hypothetical protein
28	11775	12254	+	480	160	ATG	Hypothetical protein
29	12266	12802	+	537	179	ATG	NHN homing endonuclease
30	12789	13571	+	783	261	TTG	Hypothetical protein
31	13676	14062	+	387	129	ATG	HNH homing endonuclease
32	14064	14219	+	153	52	ATG	Hypothetical protein
33	14209	15027	+	819	273	ATG	Hypothetical protein
34	15028	15276	+	249	83	ATG	Hypothetical protein
35	15360	16067	+	708	236	ATG	Hypothetical protein
36	16137	16361	+	225	75	ATG	Hypothetical protein
37	16396	18687	+	2292	764	ATG	Hypothetical protein
38	18665	19090	+	426	142	GTG	Hypothetical protein
39	19136	19360	+	225	75	ATG	Glutaredoxin
40	20430	19444	–	987	329	ATG	Phage lysin, N-acetylmuramoy-L-alanine amidase
41	20663	20427	–	237	79	ATG	Phage lysis modules
42	20922	20677	–	246	82	ATG	Phage tail fiber
43	23419	21110	–	2310	770	ATG	Phage tail assembly protein
44	25515	23431	–	2085	695	ATG	Phage protein
45	29967	25597	–	4371	1457	ATG	Phage tail tape measure
46	30535	30224	–	312	104	ATG	Phage protein
47	31287	30727	–	561	187	ATG	Phage major tail protein
48	31731	31366	–	366	122	ATG	Phage protein
49	32135	31728	–	408	136	ATG	Phage protein
50	32135	31728	–	336	112	ATG	Phage protein
51	32735	32439	–	297	99	ATG	Phage DNA packaging protein
52	32979	32779	–	201	67	ATG	Hypothetical protein
53	34339	33104	–	1236	412	ATG	Phage major capsid protein of Caudovirales
54	34972	34409	–	564	188	ATG	Phage maturation protease
55	36110	34965	–	1146	382	ATG	Phage portal (connector) protein
56	36110	34965	–	165	55	ATG	Phage protein
57	38073	36349	–	1725	575	ATG	Phage terminase, large subunit
58	38307	38188	–	120	40	ATG	Hypothetical protein
59	38795	38322	–	474	158	ATG	Phage termina, small subunit
60	39133	38798	–	336	112	ATG	Putative endodeoxyribonuclease
61	39645	39133	–	513	171	ATG	HNH homing endonuclease
62	39875	39762	–	114	38	ATG	Phage protein

### Phylogenetics

The relatedness of phiNASRA1 was assessed against 32 publicly-available *Enterococcus* phage genomes (Table S1). The phages were isolated over 12 years (2007 to 2019) from wastewater and sewage collected from municipal, hospital, and agricultural environments spanning 9 countries (Canada, China, Germany, Israel, Japan, Norway, United Kingdom, United States, and South Korea). The size of the genomes ranged from 30,505 bp (EF62phi, human, Norway, 2010) to 147,589 bp (EFDG1, sewage, Israel, 2014) with a mean of 54,523 bp. The GC content ranged from 32.7% (EF62phi, human, Norway, 2010) to 40.4% (BC611, unknown source, United States, 2011) with a mean of 36.0%. The number of ORFs ranged from 44 (SAP6, sewage, South Korea, 2011) and 221 (phiEF24C, water, Japan, 2007) with a mean of 84.

High sequence divergence prevented the inclusion of EFLK1 (*E*-value 4.40) in the large subunit terminase phylogeny. Likewise, high sequence divergence prevented the inclusion of EF62phi in the large subunit terminase and portal protein phylogenies (*E*-value 0.82 and 1.20, respectively). The length of the trimmed large subunit terminase protein sequence alignment (*N* = 30 sequences) was 721 amino acids and it was comprised of 128 constant sites and 517 parsimony informative sites with 656 distinct site patterns. The length of the trimmed portal protein sequence alignment (*N* = 31 sequences) was 690 amino acids and it was comprised of 110 constant sites and 535 parsimony informative sites with 643 distinct site patterns. For the large subunit terminase alignment, the best-fit model, according to the BIC scores and weights, was calculated as VT+F+I. For the portal protein alignment, the best-fit model was calculated as VT+F+G4.

**Figure 4:**
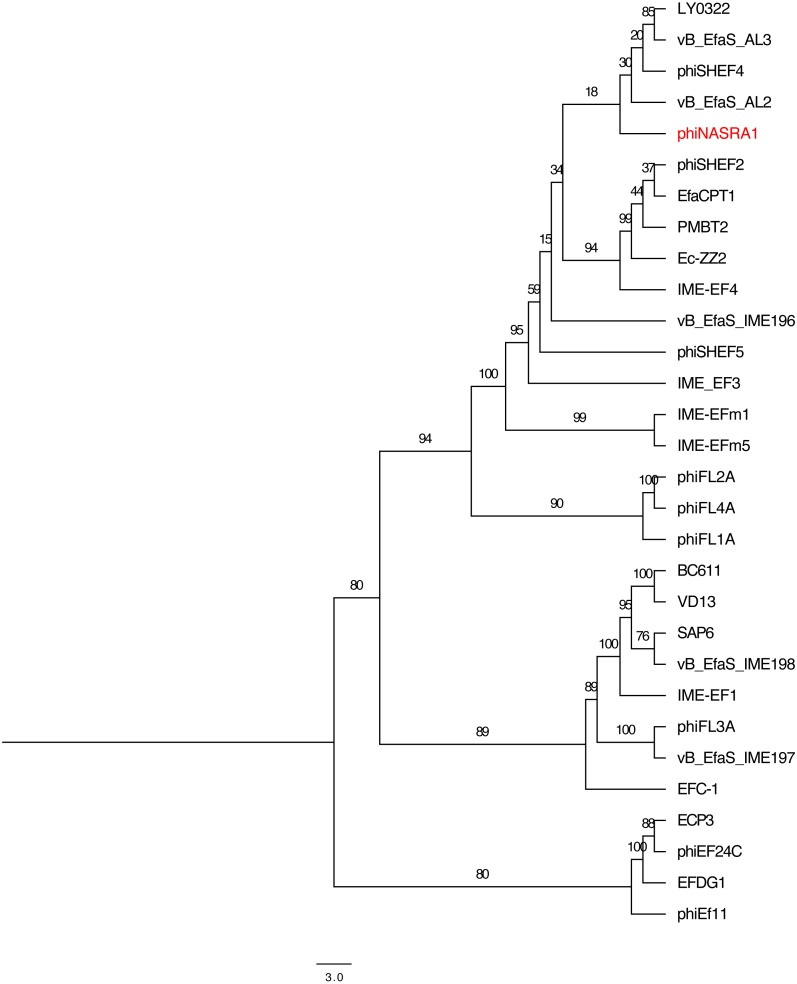
Large subunit terminase tree. Single-locus**** phylogenetic tree, based on the large subunit terminase protein, showing the relatedness of 33 *Enterococcus* phage including phiNASRA1 (shown in red font). Branch lengths represent the average number of substitutions per site and branch labels show the bootstrap support values. The tree was rooted to the midpoint.

**Figure 5:**
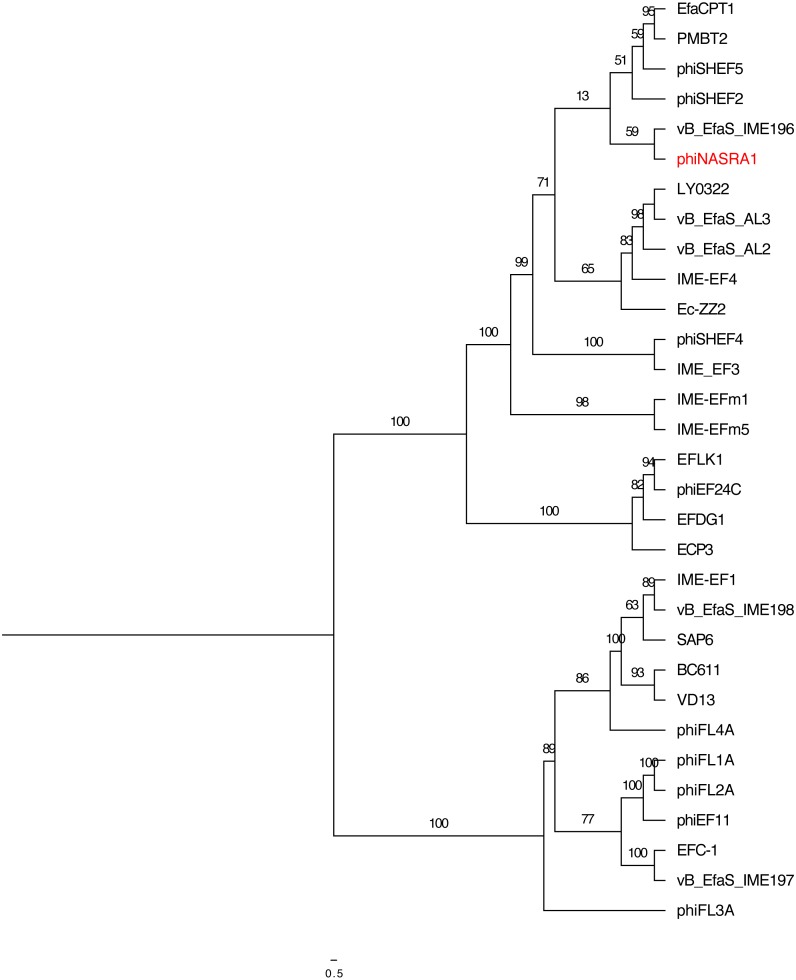
Portal tree. Single-locus phylogenetic tree, based on the portal protein, showing the relatedness of 35 *Enterococcus* phage including phiNASRA1 (shown in red font). Branch lengths represent the average number of substitutions per site and branch labels show the bootstrap support values. The tree was rooted to the midpoint.

The two single-locus phylogenies (Figs. 4 and 5, respectively) shared several features; however, the Robinson-Foulds distance could not be calculated as the trees contained different numbers of taxa. Genomes larger than 100 kbp (ECP3, EFLK1, EFDG1, and phiEF24C) formed distinct clades in both phylogenies. Both phylogenies also grouped genomes into 4 subclades: (1) BC-611, IME-EF1, vB_EfaS_IME198, SAP6, and VD13, (2) LY0322, vB_EfaS_AL2, and vB_EfaS_AL3, (3) EfaCPT1, PMBT2, and phiSHEF2, and 4) IME_EFm1 and IME_EFm5. The genomes in the first subclade were similar in size (53,996 to 58,619 bp) and GC content (40.0 to 40.4%) with one exception (vB_EfaS_IME198, 35.0%). The genomes in the second subclade were similar in provenance, size, and GC content (China, 40,789 to 40,934 bp, and 34.5 to 34.8%, respectively). Likewise, the genomes in the third subclade were similar in size and GC content (40,923 to 41,712 bp, and 34.6 to 34.7%, respectively) while the genomes in the fourth subclade were similar in provenance, size, and GC content (China, 42,265 to 42,597 bp, and 35.2 to 35.5%, respectively). The likelihood of the portal protein phylogeny was higher given the tree’s lower AIC and BIC scores and higher bootstrap values. In the large subunit terminase phylogeny, phiNASRA1 grouped with four seemingly unrelated genomes: vB_EfaS_AL2 and vB_EfaS_AL3 (hospital sewage, China, 2018), LY0322 (unknown source, China, 2019), and phiSHEF4 (wastewater, United Kingdom, 2017. In the portal protein phylogeny, phiNASRA1 grouped with only vB_EfaS_IME196 (hospital sewage, China, 2015).

### Pangenome analysis

The 32 publicly-available *Enterococcus* phage genomes (Table S1) were included in the pangenome analysis. The 32 genomes contained a total of 2,752 ORFs and the pangenome grouped those sequences into 513 homologous clusters but no sequences were homologous (based on default search settings) across all 32 genomes due to a high level of sequence diversity. Relaxing the search constraints decreased bootstrap support in the pangenome phylogeny. Among the 513 clusters, the most ubiquitous cluster encoded a glutaredoxin-like protein that was homologous in only 21/32 (65.6%) genomes. Two additional clusters (encoding hypothetical proteins) were present in at least 50% of the genomes. The large subunit terminase, portal protein, and major tail protein were homologous in only 15/32 (46.8%) of the genomes. On average, most clusters were homologous in no more than five genomes.

**Figure 6:**
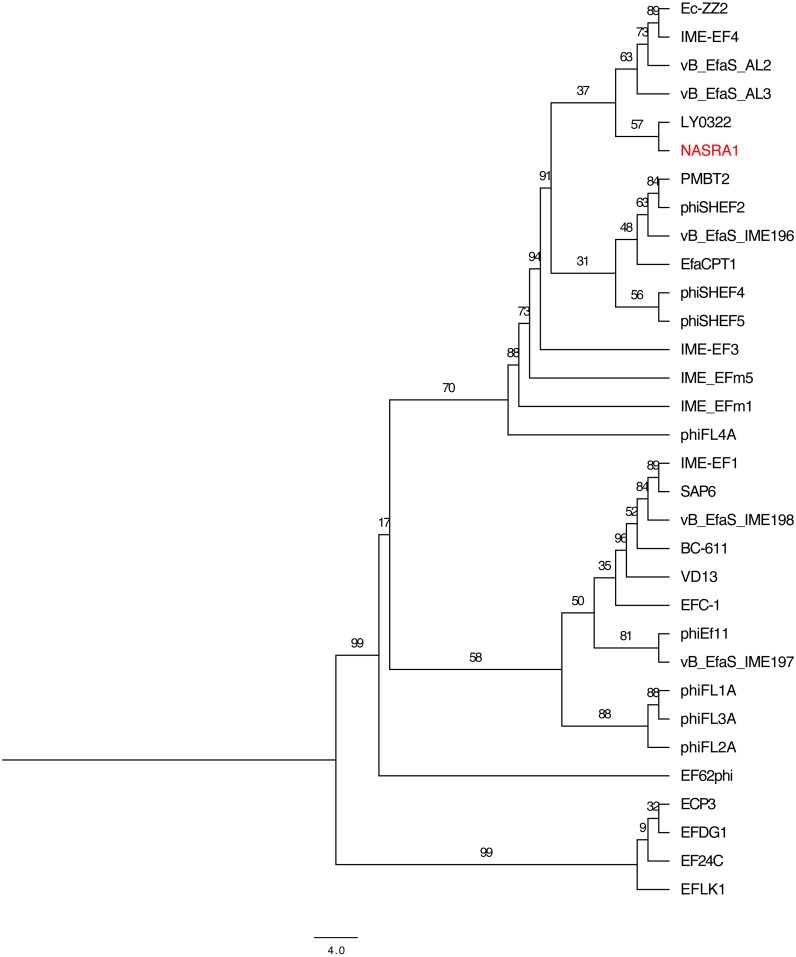
Pangenome tree. Pangenome-scale phylogenetic tree showing the relatedness of 38 *Enterococcus* phage including phiNASRA1 (shown in red font). Branch lengths represent the average number of substitutions per site and branch labels show the bootstrap support values. The phylogeny was rooted to the outgroup formed by the four genomes larger than 100 kbp (ECP3, EFLK1, EFDG1, and phiEF24C).

The pangenome phylogeny included all 32 genomes (Fig. 6). The binary presence/absence matrix contained 513 binary sites, 0 constant sites, 513 parsimony informative sites, and 137 distinct site patters. The best fit model was GTR2+F0+ASC+G4 according to the BIC scores and weights. The likelihood of the pangenome phylogeny was lower than the above single-locus phylogenies given the tree’s higher AIC and BIC scores and lower bootstrap values. The four largest genomes (ECP3, EFLK1, EFDG1, and phiEF24C) formed a distinct subclade. Additional phylogenetic subclades included subclades 1, 2, 3, and 4 (described above) although agreement between the two single-locus phylogenies was higher. In this pangenome phylogeny, phiNASRA1 grouped with LY0322 (unknown source, China, 2019) that was similar in size, GC content, and number of ORFs (40,139 and 40,934 bp, 34.77 and 34.80%, and 62 and 64 ORFs, respectively).

## Discussion

In the past three decades, antibiotic-resistant strains of *Enterococcus* have been increasingly associated with nosocomial infections and have increasingly exhibited high-level antibiotic resistance as well as multidrug resistance (Lebreton et al., 2013). The development of alternative strategies, like phage therapy, may play a critical role in combating antibiotic-resistant *Enterococcus* infections (CDC, 2013; WHO, 2014). However, knowledge of *Enterococcus* phage biology and diversity is limited. To address this knowledge gap, we isolated and characterized a phage (phiNASRA1) capable of infecting an HLAR *E. faecalis* strain, and we assessed the diversity of publicly-available phages capable of infecting this species.

The provenance of phiNASRA1 mirrored that of the 32 publicly available *Enterococcus* phage genomes (see Table S1), as most were isolated from wastewater or sewage. Among these, phages phiSHEF2, phiSHEF4, and phiSHEF5 were effective at eradicating antibiotic-resistant *E. faecalis* endodontic infections (Zubidi Al, 2017). In contrast, the therapeutic potential of IME-EF3 and EFRM31 was questioned as the former was shown to carry a metallo-beta-lactamase gene responsible for multidrug resistance (Li et al., 2014) and the latter carries a ABC transporter shown to play a role in the transduction of gentamicin resistance (Fard et al., 2010). In this study, the high lytic efficiency of phiNASRA1 and the absence of genes associated with drug resistance were encouraging but further testing is needed to determine the phage’s ability to clear an antibiotic-resistant infection in an animal model.

Phages commonly exhibit a narrow host range and previous studies have shown that phages infecting *Enterococcus* can be highly specific (Purnell, Ebdon & Taylor, 2011). By contrast, previous studies have also shown that *Enterococcus* phages are capable of infecting both *E. faecalis* and *E. faecium* (Zhang et al., 2013). Here, host specificity testing showed that phiNASRA1 was capable of infecting a minority (*N* = 3∕20) of the strains tested, which suggests a narrow host range. However, a definitive conclusion about host specificity cannot be reached without testing a larger and more diverse strain collection under a range of physiological conditions. Previous studies have shown that the use of multiple hosts during isolation can improve the likelihood of isolating a phage with a broad host range (Ross, Ward & Hyman, 2016).

Previous studies have assessed the relatedness of *Enterococcus* phages by constructing phylogenies based on the large subunit terminase and portal proteins (Wang et al., 2014). In this study, these proteins were valuable phylogenetic markers that grouped some genomes by provenance, size, and GC content. The strongest feature of the two phylogenies was that phages larger than 100 kbp grouped together despite stark differences in provenance. A previous study reported a clear phylogenetic distinction between small and large *Aeromonas* phages (Vincent et al., 2017). That study also reported a clear divergence in GC content between small and large phage genomes but a size-based trend in GC content was not observed in this study. Rather, variations in *Enterococcus* phage GC content (range 32.7 to 40.4%, *N* = 32 phages) closely mirrored the GC content of publicly available hosts (range 35.8 to 41.0%, *N* = 1,428 *E. faecalis* genomes in the NCBI database).

Tettelin et al. (2005) proposed that the bacterial genome is comprised of essential (i.e., core) and accessory genes. Core genes would be shared by all members of a species while accessory genes could be unique to each community or individual. Here, the high degree of sequence diversity increased the difficulty of finding homologous sequences across the 32 *Enterococcus* phage and made clear that the concept of a core genome may not apply to some phages. That only six coding sequences were homologous in half of the phages and the majority of coding sequences were homologous in no more than five phages demonstrates the high degree of sequence diversity. A pangenome analysis of *Aeromonas* phages showed that phage forming distinct phylogenetic groups possess a robust core genome (Vincent et al., 2017). The same was true in this study when analyzing the pangenome of the four larger *Enterococcus* phage that shared 63 homologous gene clusters. Thus, in restricted scenarios, such as the pangenome analysis of larger *Enterococcus* phage phage that form phylogenetically distinct groups, the concept of a core genome may be applicable.

## Conclusions

The discovery and characterization of phiNASRA1 contributes to a growing body of literature devoted to understanding phage biology and diversity. The biology of the phage (e.g., high lytic efficiency and the absence of genes associated with drug resistance) suggest that phiNASRA1 may have therapeutic potential, athough the potential is diminished by the narrow host range and further work is needed to test its ability to eradicate antibiotic-resistant *E. faecalis* infections in an animal model. Phylogenetic relationships were easier to elucidate using single-locus phylogenies compared to the pangenome phylogeny. Regardless, the pangenome analysis clearly illustrated the extent of sequence divergence across the 32 genomes and therefore, advanced our understanding of *Enterococcus* phage diversity and genome plasticity.

##  Supplemental Information

10.7717/peerj.9171/supp-1 Supplemental Information 1 Description of the 38 *Enterococcus* phage genomes included in the phylogenetic and pangenomic analyses

10.7717/peerj.9171/supp-2 Supplemental Information 2 Raw data for the one-step growth curve shown in Fig. 3

## Additional Information and Declarations

### DNA Deposition

The following information was supplied regarding the deposition of DNA sequences: The original phage genome assembly and annotation are publicly available at NCBI: MG264739.

### Data Availability

The following information was supplied regarding data availability: The raw data supporting the one-step growth curve in Fig. 3 is available as a Supplemental File.
