# Supplementary material for: Isolation, characterization, and comparative genomic analysis of a phage infecting high-level aminoglycoside-resistant (HLAR) Enterococcus faecalis
Source: PeerJ. 2020 May 21;8:e9171. doi: 10.7717/peerj.9171 (PMC7246028; doi:10.7717/peerj.9171)
Supplement: Supplemental Information 1 [file peerj-08-9171-s001.docx]

**Table S1.** Description of the 32 *Enterococcus* phage genomes included in the phylogenetic and pangenomic analyses.

| **Phage** | **Host** | **Source^a^** | **Provenance** | **Size (bp)** | **%GC** | **ORFs** | **Accession #** |
| --- | --- | --- | --- | --- | --- | --- | --- |
| BC-611 | *E. faecalis* | unknown | United States, 2012 | 53,996 | 40.4 | 87 | NC_018086 |
| Ec-ZZ2 | *E. faecalis* | hospital sewage | China, 2015 | 41,170 | 34.6 | 59 | NC_031260 |
| ECP3 | *E. faecalis* | unknown | South Korea, 2019 | 145,518 | 35.9 | 220 | NC_027335 |
| EF62phi | *E. faecalis* | human | Norway, 2010 | 30,505 | 32.7 | 48 | NC_017732 |
| EfaCPT1 | *E. faecalis* | sewage | United States, 2012 | 40,923 | 34.6 | 69 | NC_025465 |
| EFC-1 | *E. faecalis* | unknown | South Korea, 2018 | 40,286 | 35.0 | 59 | NC_025453 |
| EFDG1 | *E. faecalis* | sewage | Israel, 2014 | 147,589 | 37.2 | 192 | NC_029009 |
| EFLK1 | *E. faecalis* | sewage | Israel, 2014 | 130,952 | 35.9 | 198 | NC_029026 |
| IME_EF1 | *E. faecalis* | sewage | China, 2012 | 57,081 | 40.0 | 98 | NC_041959 |
| IME-EF3 | *E. faecalis* | sewage | China, 2013 | 41,687 | 34.6 | 69 | NC_023595 |
| IME-EF4 | *E. faecalis* | sewage | China, 2013 | 40,692 | 34.6 | 60 | NC_023551 |
| IME-EFm1 | *E. faecium* | hospital sewage | China, 2014 | 42,597 | 35.2 | 69 | NC_024356 |
| IME-EFm5 | *E. faecalis* | sewage | China, 2015 | 42,265 | 35.5 | 70 | NC_028826 |
| LY0322 | *E. faecalis* | unknown | China, 2015 | 40,934 | 34.8 | 64 | NC_042125 |
| phiEf11 | *E. faecalis* | human | United States, 2009 | 42,822 | 34.4 | 65 | NC_013696 |
| phiEF24C | *E. faecalis* | water | Japan, 2007 | 142,072 | 35.7 | 221 | NC_009904 |
| phiFL1A | *E. faecalis* | human | United Kingdom, 2010 | 38,764 | 34.0 | 61 | NC_013646 |
| phiFL2A | *E. faecalis* | human | United Kingdom, 2010 | 36,270 | 34.6 | 63 | NC_013643 |
| phiFL3A | *E. faecalis* | human | United Kingdom, 2010 | 39,576 | 34.5 | 64 | NC_013648 |
| phiFL4A | *E. faecalis* | human | United Kingdom, 2010 | 37,856 | 37.8 | 55 | NC_013644 |
| phiNASRA1 | *E. faecalis* | wastewater | United States, 2017 | 40,139 | 34.7 | 62 | MG264739 |
| phiSHEF2 | *E. faecalis* | wastewater | United Kingdom, 2017 | 41,712 | 34.6 | 68 | NC_042021 |
| phiSHEF4 | *E. faecalis* | wastewater | United Kingdom, 2017 | 41,081 | 34.7 | 63 | NC_042022 |
| phiSHEF5 | *E. faecalis* | wastewater | United Kingdom, 2017 | 41,598 | 34.7 | 69 | NC_042023 |
| PMBT2 | *E. faecalis* | sewage | Germany, 2017 | 41,489 | 34.7 | 67 | NC_042101 |
| SAP6 | *E. faecalis* | sewage | South Korea, 2011 | 58,619 | 40.0 | 44 | NC_041960 |
| vB_EfaS_AL2 | *E. faecalis* | hospital sewage | China, 2018 | 40,836 | 34.5 | 62 | NC_042127 |
| vB_EfaS_AL3 | *E. faecalis* | hospital sewage | China, 2018 | 40,789 | 34.8 | 61 | NC_042126 |
| vB_EfaS_IME196 | *E. faecalis* | hospital sewage | China, 2015 | 38,886 | 35.0 | 57 | NC_028990 |
| vB_EfaS_IME197 | *E. faecalis* | hospital sewage | China, 2015 | 41,098 | 34.1 | 67 | NC_028671 |
| vB_EfaS_IME198 | *E. faecalis* | hospital sewage | China, 2015 | 58,000 | 35.0 | 95 | NC_029016 |
| VD13 | *E. faecalis* | urogenital secretion | Canada, 2018 | 55,726 | 40.0 | 88 | NC_024212 |

^a^Source of isolation given if included in GenBank
